# Relationship between vestibular alterations and cognitive performance in adults and older adults

**DOI:** 10.1590/2317-1782/e20240356en

**Published:** 2026-04-27

**Authors:** Tâmara Isis Santana Santos Barreto, Jeferson Sampaio d’Ávila, Suyanne dos Santos Mendonça, Raphaela Barroso Guedes Granzotti, Ademir Antônio Comerlatto, Josilene Luciene Duarte, Mariane Perin da Silva Comerlatto, Marina Panelli, Luciane Domingues Figueiredo Mariotto, Kelly da Silva

**Affiliations:** 1 Programa de Pós-graduação em Ciências Aplicadas à Saúde, Universidade Federal de Sergipe – UFS - Lagarto (SE), Brasil.; 2 Otocenter – Clínica de Otorrinolaringologia Prof. Dr. Jeferson d’Ávila - Aracaju (SE), Brasil.; 3 Curso de Fonoaudiologia, Universidade Federal de Sergipe – UFS - Lagarto (SE), Brasil.; 4 Interacoustics Brasil - São Paulo (SP), Brasil.; 5 Faculdade de Odontologia de Bauru, Universidade de São Paulo – USP - Bauru (SP), Brasil.

**Keywords:** Vestibular System, Postural Balance, Cognition, Dizziness, Educational Status

## Abstract

**Purpose:**

To analyze the influence of vestibular changes on the cognitive performance of adults and elderly people, with 11 or more years of study.

**Methods:**

Observational, cross-sectional, and quantitative study that evaluates the vestibular system and cognitive profile of adults and elderly individuals with 11 or more years of study. The balance assessment consisted of the Head Impulse Test video examination and positioning maneuvers (Dix-Hallpike and Head Roll Test). Cognition was assessed using the Brief Neuropsychological Assessment Instrument – NEUPSILIN. The data were analyzed descriptively and inferentially, through a multiple linear regression using the SPSS 25.0 software, and a significance level of 5% was considered.

**Results:**

Study carried out with 60 participants. An association was observed between changes in vHIT and positive Dix Hallpike, which were related to worse performance in Verbal Memory. Increasing age was associated with worse performance in Working Memory and Long-Term Memory.

**Conclusion:**

The presence of vestibular alterations was related to worse cognitive performances of adults and elderly people in tests of temporal-spatial orientation, attention, perception, arithmetic skills and verbal memory. Increasing age was associated with worse performance in working memory and long-term memory.

## INTRODUCTION

The primary function of the vestibular system relates to the maintenance of gaze stability and balance through reflex mechanisms^(1)^. This system detects angular and linear head acceleration in three dimensions and generates the vestibulo-ocular reflex (VOR) and the vestibulospinal reflex (VSR), which stabilize the visual image on the retina and adjust posture, respectively, during head movements. Additionally, the visual, somatosensory, and proprioceptive systems contribute to this function^(2-4)^.

Vestibular dysfunction is a health condition that compromises activities of daily living and results in significant impairment in quality of life. Common symptoms include vertigo, dizziness, gait disturbances, falls, hearing loss, and tinnitus^(5,6)^.

In recent decades, the vestibular system has been viewed not only as a reflexive system but has also been associated with cognitive performance^(1,7-11)^. In general, studies indicate that bilateral vestibular lesions lead to more severe cognitive deficits than unilateral dysfunctions^(4,5,9)^. The cognitive perspective relates to cortical cells that integrate knowledge about actions and perceptions not only as mechanisms for integrated action and perceptual processing but also as a neural substrate that supports a wide range of higher cortical functions, including attention, meaning, and concepts, goals, sequences, and intentions^(12)^_._

Laboratory and clinical findings indicate an association between vestibular inputs and a variety of higher-order functions, particularly memory, as this domain is also influenced by other cognitive processes such as attention, emotional disturbances, and executive functions^(13)^.

Bigelow and Agrawal^(1)^ reviewed studies linking vestibular dysfunction to impairments in visuospatial ability, attention, memory, and executive function. Studies addressing cognitive aspects in individuals with vestibular disorders or complaints often encounter participants’ educational level^(14-18)^ as a confounding factor, which may influence the results or limit their generalizability. Considering this issue, the present study included only individuals with at least eleven years of formal education to examine the relationship between the vestibular system and cognitive performance in adults and older adults.

Thus, this study seeks to clarify the relationship between vestibular alterations and cognitive performance in educated adults and older adults. Given that individuals with vestibular disorders may present some degree of cognitive impairment, this study aims to analyze whether peripheral-level vestibular alterations can lead to central-level changes (cognitive and memory impairments) and to examine the relationship between these functions in contexts of cognitive development favored by educational attainment.

## METHODS

This study is cross-sectional observational with a quantitative, analytical approach based on individual-level observations. This study was conducted strictly in accordance with national and international ethical guidelines for research involving human subjects and was approved by a Research Ethics Committee (approval number 4,857,581).

It included a convenience sample of 60 participants aged 19 years or older, of any gender, with 11 or more years of formal education, and hearing within normal limits. To ensure that all participants had normal hearing, an audiometry was performed as an inclusion criterion, given that hearing impairment may affect the comprehension of cognitive assessment instructions and therefore lead to poorer task performance. Hearing threshold classification followed the criteria proposed by Lloyd and Kaplan^(19)^. The study recruited all participants from the Audiology Department of a private clinic in the city of Aracaju, Sergipe, and all had referrals for the examinations.

Were excluded participants with genetic syndromes; those undergoing chemotherapy; individuals diagnosed with dementia or with previous diagnoses of cognitive impairment; those diagnosed with attention-deficit/hyperactivity disorder; individuals with a history of stroke; those using anxiolytic, antidepressant, anticonvulsant, or sedative medications; individuals with hearing loss of any type or degree; and those who had undergone vestibular rehabilitation at any time.

Participants underwent the following procedures: speech-language anamnesis, the *video Head Impulse Test* (vHIT), *Dix-Hallpike* and *Head Roll Test* positioning maneuvers, and cognitive assessment using the NEUPSILIN Brief Neuropsychological Assessment Instrument.

The researcher administered the speech-language anamnesis, which included information on the participants’ general health, audiological and otoneurological data, as well as occupational activity.

The otoneurological assessment was divided into two stages: the *vHIT* and the diagnostic positioning maneuvers, including the *Dix-Hallpike* and *Head Roll Test*. The *Dix-Hallpike* ^(20)^ maneuver evaluates otolith displacement through changes in head position and shows greater sensitivity for the anterior and posterior semicircular canals. The maneuver was performed with the patient seated on an examination table; the head was rotated 45° toward the ear being tested, and the patient was then positioned supine with the head extended approximately 20° beyond the edge of the head support. During this, the participant was instructed to keep their eyes open to allow observation of possible nystagmus. When nystagmus was observed, the maneuver was considered positive. When not observed, the maneuver was considered negative.

The *Head Roll Test* ^(21)^ aimed to assess otolith displacement through changes in head position and shows greater sensitivity for the lateral semicircular canals. The test was performed with the participant in the supine position on an examination table, with the head elevated at 30°, allowing a 90° rotation toward the ear being examined and enabling observation of possible nystagmus. During the maneuver, the participant was instructed to keep their eyes open. The maneuver was considered positive in the presence of nystagmus and negative in its absence.

The vHIT^(22)^ assesses the vestibulo-ocular reflex in each semicircular canal individually at the physiological frequency of angular head acceleration by means of rapid, short-amplitude head impulses. This examination used the *EyeSeeCam vHIT* from *Interacoustics*. The assessment was performed with the participant seated in a chair one meter from a visual target positioned at eye level, wearing a mask (goggles with an attached camera). Eye and head calibration was performed first, followed by assessment of the lateral semicircular canals and then the vertical semicircular canals, allowing evaluation of the synergistic pairs right anterior/left posterior and left anterior/right posterior. During the assessment, instructions were given to maintain gaze fixation on the target while the examiner delivered head impulses for the stimulation planes of the six semicircular canals. At least 15 impulses were obtained for each plane. Head movements were delivered in unpredictable directions and at unpredictable frequencies, with low amplitude (10° to 20º) and velocities ranging from 100°/s to 250°/s. For each impulse, the head movement and the reflexive eye response were recorded using a sinusoidal graph. This recording allowed calculation of the VOR gain, with normal values ranging from 0.77 to 1.33 ms^(23)^. The vHIT results were classified as normal or abnormal, whereas the Dix–Hallpike maneuver results were categorized as present or absent.

Cognitive assessment was conducted using the NEUPSILIN Brief Neuropsychological Assessment Instrument^(24)^. This instrument consists of a brief assessment battery designed to provide a neuropsychological profile (quantitative and qualitative). The instrument assesses eight main neuropsychological functions and consists of 32 tasks, with a mean administration time of approximately 50 minutes. It assesses the following abilities: temporal-spatial orientation, attention, visual perception, memory (working, verbal episodic, semantic, short-term visual, and prospective), arithmetic skills, oral and written language, praxis (ideomotor, constructive, and reflexive), and executive functions (problem-solving and phonemic-orthographic verbal fluency).

The examiner administered the assessment, and throughout the procedure, the participant remained comfortably seated, with a blank sheet of paper and a pen to take necessary notes and answer the questions as instructed.

The study used Z-scores to establish the cutoff points for indicating deficits in specific tasks or functions, as this method allows comparison of results despite differences in age and educational level among participants. According to the instrument organizers, this score should be calculated based on the individual’s raw score, the group mean for the test (task or function), and the group standard deviation for the test (task or function).

Thus, Z-scores were classified as follows: superior when above 1.0; average when between 0.99 and −0.99; alert for deficit when between −1.0 and −1.4; deficit at −1.5; moderate-to-severe deficit when between −1.6 and −1.99; and severe deficit when below −2.

Data were analyzed using descriptive and inferential statistics with SPSS version 25.0. Descriptive analysis of quantitative variables included measures of central tendency (mean and median), variability (standard deviation), and position (minimum, maximum, first quartile, and third quartile). Descriptive analysis of qualitative variables included absolute frequencies and relative percentages.

Z-scores were calculated using the following Formula 1:


Z=(X−M)/SD (1)

wherein X represents the individual’s score on the task, M represents the mean of the reference group (provided by age and educational level in the test manual), and SD represents the standard deviation of that group.

The Shapiro–Wilk normality test was conducted to determine whether to use the *T-Student* or *Mann-Whitney* test. Multiple linear regression models were used to predict the quantitative dependent variables. Independent variables were selected using the *stepwise* method. A significance level of 5% was adopted for all inferential analyses.

## RESULTS

The study included 60 participants aged 19 to 74 years, with a mean age of 49 years and three months; 40 were women (66.7%), and 20 were men (33.3%). Regarding educational level, 30 participants had completed secondary education, and 30 had completed higher education.

The *vHIT* results were abnormal in eleven participants, with the observed alterations consisting of hypofunction of the lateral semicircular canal and the posterior semicircular canal. Additionally, vHIT abnormal and normal results were analyzed according to performance on the cognitive tests (Table 1). Statistically significant differences were observed for attention, perception, and arithmetic skills, with poorer performance among individuals with abnormal vHIT results.

**Table 1:** Comparison of cognitive performance variables in participants with and without vHIT abnormalities

Cognitive ability		Statistic	Df	p
TEMPORAL-SPATIAL ORIENTATION	Mann-Whitney U	90.0		0.85
ATTENTION	Mann-Whitney U	35.5		0.01^*^
PERCEPTION	Student's t	-2.08	28.0	0.04*
WORKING MEMORY	Student's t	0.36	28.0	0.72
VERBAL MEMORY	Student's t	-0.96	28.0	0.34
LONG-TERM MEMORY	Mann-Whitney U	80.0		0.50
SHORT-TERM MEMORY	Mann-Whitney U	82.5		0.58
PROSPECTIVE MEMORY	Mann-Whitney U	90.5		0.87
ARITHMETIC SKILLS	Mann-Whitney U	47.5		0.03*
ORAL LANGUAGE	Student's t	1.32	28.0	0.20
WRITTEN LANGUAGE	Mann-Whitney U	77.5		0.45
PRAXIS	Student's t	0.04	28.0	0.97
PROBLEM-SOLVING	Mann-Whitney U	92.0		0.93
VERBAL FLUENCY	Student's t	-0.12	28.0	0.91

* Indicates statistically significant values. vHIT was altered only in participants from the Study Group (with complaints and/or vestibular alterations)

In the *Dix-Hallpike* diagnostic positioning maneuver, nine participants showed positive results, meaning the presence of nystagmus, and a significant association was observed with poorer performance in temporal-spatial orientation (Table 2). In the *Head Roll Test*, none of the participants presented nystagmus; therefore, all results were negative.

**Table 2:** Comparison of cognitive performance variables in participants, according to the response to the Dix–Hallpike maneuver

	Dix-Hallpike	No. participants	Mean	Median	SD	p-value
TEMPORAL-SPATIAL ORIENTATION	Absent	51	0.2121	0.2963	0.821	0.564
	Present	9	0.0122	0.558	1.542	0.034^*^
ATTENTION	Absent	51	79.744	70.000	2.599	0.321
	Present	9	92.410	7.000	6.822	0.756
PERCEPTION	Absent	51	-0.1750	-0.0849	1.013	0.551
	Present	9	-0.3956	-0.619	1.040	0.393
WORKING MEMORY	Absent	51	-0.1511	-0.0312	1.233	0.477
	Present	9	-0.4823	-1.058	1.543	0.597
VERBAL MEMORY	Absent	51	-0.8146	-0.9789	1.056	0.112
	Present	9	-14.265	-1.310	1.005	0.159
LONG-TERM MEMORY	Absent	51	-0.5138	0.2174	1.550	0.853
	Present	9	-0.6197	0.217	1.705	0.058
SHORT-TERM MEMORY	Absent	51	-0.0491	0.3548	1.068	0.828
	Present	9	-0.1349	0.481	1.204	0.232
PROSPECTIVE MEMORY	Absent	51	-0.7674	0.5306	1.683	0.267
	Present	9	-14.529	-2.130	1.749	0.743
ARITHMETIC SKILLS	Absent	51	-13.548	0.3158	2.488	0.314
	Present	9	-0.4922	0.316	1.110	0.941
ORAL LANGUAGE	Absent	51	-0.7054	-0.9839	1.319	0.644
	Present	9	-0.4885	-0.337	1.110	0.511
WRITTEN LANGUAGE	Absent	51	-0.9830	-0.7925	1.087	0.527
	Present	9	-0.7381	-0.353	0.906	0.755
PRAXIS	Absent	51	-13.552	-14.928	1.454	0.882
	Present	9	-14.302	-1.769	0.866	0.934
PROBLEM-SOLVING	Absent	51	-0.3997	0.3421	1.388	0.377
	Present	9	-0.8523	0.429	1.522	0.574
VERBAL FLUENCY	Absent	51	-22.948	-22.495	0.350	0.626
	Present	9	-22.306	-2.249	0.437	0.975

* Indicates differences between groups with and without nystagmus in the Dix–Hallpike test

Caption: Dix-Hallpike absent or present indicates the absence or presence of nystagmus during the maneuver; SD = standard deviation

Multiple linear regression analysis was performed to examine whether the independent variables could predict the dependent variables: attention, working memory, verbal memory, long-term memory, short-term memory, oral language, and written language. No significant regression models were identified for four dependent variables: attention, short-term memory, oral language, and written language.

The independent variable age (β = −0.283; t = −2.249; p = 0.028) significantly predicted the dependent variable working memory [F(1.58) = 5.059; p = 0.028; R^2^ = 0.064]. The negative coefficient indicates that as age increases, working memory performance decreases; thus, older age is associated with poorer performance in this cognitive function. The equation that describes this relationship is


(WORKING MEMORY)=0.846+−0.021(Age) (2)

as shown in Table 3.

**Table 3:** Association between age and working memory performance

	Unstandardized coefficients		Standardized coefficients	t	p-value	95.0% CI for B	
	B	Standard error	Beta			Lower limit	Upper limit
(Constant)	0.846	0.492		1.720	0.091	-0.138	1.831
AGE	-0.021	0.009	-0.283	-2.249	0.028	-0.040	-0.002

Multiple linear regression; stepwise method

**Caption:** IC = confidence interval

As shown in Table 4, the independent variables DIX RE (β = −0.301; t = −2.463; p = 0.016) and vHIT (β = −0.25088778114266; t = −2.050; p = 0.044) predicted the dependent variable verbal memory [F(1.57) = 5.059; p = 0.028ᵇ; R^2^ = 0.0643]. Similarly, vHIT also showed a negative coefficient (B = −0.684; p = 0.045), suggesting that alterations in the vestibulo-ocular reflex are associated with reduced performance in this cognitive function. The equation that describes this relationship is

**Table 4:** Association between vestibular alterations (Dix–Hallpike OD and vHIT) and verbal memory performance

	Unstandardized coefficients		Standardized coefficients	t	p-value	95.0% CI for B	
	B	Standard error	Beta			Lower limit	Upper limit
(Constant)	-0.666	0.151		-4.410	0.000	-0.968	-0.363
DIX RE	-0.990	0.402	-0.301	-2.463	0.017	-1.795	-0.185
VHIT	-0.684	0.333	-0.251	-2.051	0.045	-1.351	-0.016

Multiple linear regression; stepwise method

**Caption:** CI = confidence interval; vHIT = Head Impulse Test; DIX = Dix–Hallpike maneuver; RE = right ear


$$\begin{array}{c} \text{(VERBAL MEMORY})=-0.665+\\ -0.989(\text{DIX RE)}+-0,683(\text{VHIT)} \end{array}$$ (3)

Table 5 shows that the independent variable Age (β = 0.358; t = 2.928; p = 0.004) predicted the dependent variable Long-term Memory [F(1.58) = 8.574; R^2^ = 0.113]. The equation that describes this relationship is

**Table 5:** Association between age and long-term memory performance

	Unstandardized coefficients		Standardized coefficients	t	p-value	95.0% CI for B	
	B	Standard error	Beta			Lower limit	Upper limit
(Constant)	-2.152	0.586		-3.675	0.001	-3.324	-0.980
AGE	0.033	0.011	0.359	2.928	0.005	0.010	0.055

Multiple linear regression; *stepwise* method

**Caption:** CI = confidence interval


(LONG-TERM MEMORY)=−2.151+0.032(Age) (4)

## DISCUSSION

The results of this study demonstrate a significant association between vestibular alterations, specifically hypofunction of the lateral and posterior semicircular canals observed on vHIT, the presence of nystagmus in the Dix–Hallpike test, and impairment of cognitive functions in adults and older adults. These findings reinforce the literature highlighting the role of the vestibular system not only in the regulation of balance and posture but also as a modulator of cognitive performance, with potential impact on domains such as memory and attention.

The literature indicates a higher prevalence of dizziness with increasing chronological age, with a peak between 46 and 55 years^(25-27)^. In our sample, the mean age of the participants was 49 years and three months, corroborating the findings of the cited studies.

As this study evaluates cognitive aspects, participants’ educational level may act as a factor that favors biased results, since some theories suggest differences in brain connectivity between literate and illiterate individuals; as higher educational levels are associated with greater cognitive reserve, increased synaptic density, and enhanced cerebral vascularization. They further argue that educational attainment is one of the factors that can protect against cognitive decline^(28-30)^.

National and international studies have shown that educational level influences performance on verbal and nonverbal neuropsychological tasks, as well as on memory, attention, language, and executive function tasks^(14-18,31)^. In this study, educational level does not represent a limitation, as participants were matched according to their educational attainment. Furthermore, all participants had more than 11 years of formal education. Studies involving participants with this level of education may contribute to understanding cognition without the bias associated with low educational attainment, thereby allowing a more direct examination of the influence of vestibular alterations on cognitive performance, which represents a major strength of the present study.

Cognitive performance was influenced by the alterations identified in the *vHIT* and *Dix-Hallpike* tests, as well as age. Abnormal *vHIT* results and a positive *Dix-Hallpike* test indicate vestibular dysfunction and were associated with performance in temporal-spatial orientation, attention, perception, arithmetic skills, and verbal memory.

Abnormal *vHIT* results were statistically significant for attention, perception, and arithmetic skills. Thus, when *vHIT* results were abnormal, performance in these functions was poorer. Studies by Bessot et al.^(32)^ and Popp et al.^(33)^ also showed poorer performance in attention among participants with vestibular dysfunction.

No clear relationship between vestibular dysfunction and poorer performance in arithmetic skills has been reported in the literature, which may encourage further research^(34-36)^. However, the literature shows a relationship between poorer performance in arithmetic skills observed in populations with attention deficits, which may be related to the findings of this study^(37-39)^.

Temporal-spatial orientation was also associated with poorer performance in participants with vestibular dysfunction, due to the presence of nystagmus in the *Dix-Hallpike* maneuver. Studies by Hufner et al.^(40)^, Guidetti et al.^(41)^, Xie et al.^(42)^, Ahmad et al.^(43)^, and Chari et al.^(11)^ corroborated this finding. Brandt et al.^(44)^ showed a direct relationship between hippocampus volume and spatial orientation. The study was conducted using magnetic resonance imaging volumetry, which showed that participants with bilateral vestibular dysfunction developed significant hippocampus atrophy (an average reduction of 16.9%) and exhibited deficits in spatial orientation compared to the control group.

In this study, verbal memory was poorer in individuals with abnormalities in the *vHIT* and/or the *Dix-Hallpike* maneuver. Caixeta et al.^(45)^ found the same relationship through the assessment of 76 patients with chronic peripheral vestibular dysfunction. However, the vestibular assessment tools were not the same as those used in the present study, and for cognitive evaluation, they employed screening tests (the Mini-Mental State Examination, the Clock Drawing Test, and the Verbal Fluency Test). Thus, the present study provides a more comprehensive assessment of cognitive performance, given that the full battery of tests comprising NEUPSILIN was used, rather than just *screening* tests.

Age showed an influence on long-term and working memory, such that higher age was associated with poorer performance in these functions, which is consistent with numerous national and international studies^(31,46,47)^. A study involving 1,126 participants aged 60 to 100 years examined the effect of age on memory. The authors used the *Mini-Mental State Examination* which assesses working memory; the *Montreal Cognitive Assessment*, which evaluates verbal declarative memory; and the Rey–Osterrieth Complex Figure Test, which assesses visuospatial memory. Thus, they observed that age influences all types of memory assessed, partially corroborating our finding that age affects working memory^(48)^. Participant educational level may have contributed to smaller impairments in other types of memory, as all had at least 11 years of formal education.

One of the limitations of this study relates to the sample size, which was a convenience sample, thereby limiting the generalizability of the results to the broader population. Therefore, future studies with larger and more representative samples are recommended in order to allow broader inferences about the Brazilian population.

## CONCLUSION

The presence of vestibular alterations was associated with poorer cognitive performance in adults and older adults on tests of temporal-spatial orientation, attention, perception, arithmetic skills, and verbal memory. Increasing age was associated with poorer performance in working memory and long-term memory.
