# Supplementary material for: Relationship between vestibular alterations and cognitive performance in adults and older adults
Source: Codas. 2026 Apr 27;38(3):e20240356. doi: 10.1590/2317-1782/e20240356en (PMC13143033; doi:10.1590/2317-1782/e20240356en)
Supplement: Translation

# Translation: Relação entre as alterações vestibulares e o desempenho cognitivo de adultos e idosos

**DOI:** 10.1590/2317-1782/e20240356pt

**Authors:** Tâmara Isis Santana Santos Barreto, Jeferson Sampaio d’Ávila, Suyanne dos Santos Mendonça, Raphaela Barroso Guedes Granzotti, Ademir Antônio Comerlatto, Josilene Luciene Duarte, Mariane Perin da Silva Comerlatto, Marina Panelli, Luciane Domingues Figueiredo Mariotto, Kelly da Silva

**Affiliations:** 1 Programa de Pós-graduação em Ciências Aplicadas à Saúde, Universidade Federal de Sergipe – UFS - Lagarto (SE), Brasil.; 2 Otocenter – Clínica de Otorrinolaringologia Prof. Dr. Jeferson d’Ávila - Aracaju (SE), Brasil.; 3 Curso de Fonoaudiologia, Universidade Federal de Sergipe – UFS - Lagarto (SE), Brasil.; 4 Interacoustics Brasil - São Paulo (SP), Brasil.; 5 Faculdade de Odontologia de Bauru, Universidade de São Paulo – USP - Bauru (SP), Brasil.

**Keywords:** Sistema Vestibular, Equilíbrio Postural, Cognição, Tontura, Escolaridade

## Abstract

**Objetivo:**

Analisar a relação entre as alterações vestibulares e o desempenho cognitivo de adultos e idosos, com 11 anos ou mais de escolaridade.

**Método:**

Estudo observacional, transversal e quantitativo que avaliou o sistema vestibular e o perfil cognitivo de pessoas adultas e idosas, com 11 ou mais anos de estudo. A avaliação do equilíbrio foi composta pelo exame vídeo Head Impulse Test (vHIT) e manobras de Dix-Hallpike e Head Roll Test. O perfil cognitivo foi avaliado por meio do Instrumento de Avaliação Neuropsicológica Breve NEUPSILIN. Os dados foram analisados de forma descritiva e inferencial, por meio de uma regressão linear múltipla, utilizando-se o software SPSS 25.0 sendo considerado o nível de significância de 5%.

**Resultados:**

Estudo realizado com 60 participantes. Observou-se associação entre a alteração no vHIT e no Dix Hallpike positivo, que se mostraram relacionados a pior desempenho na Memória Verbal. O aumento da idade esteve associado a piores desempenhos na Memória de Trabalho e na Memória de Longo Prazo.

**Conclusão:**

A presença de alterações vestibulares esteve relacionada à piores desempenhos cognitivos de adultos e idosos nas provas de orientação temporoespacial, atenção, percepção, habilidades aritméticas e memória verbal. O aumento da idade foi associado ao pior desempenho na memória de trabalho e na memória de longo prazo.

## INTRODUÇÃO

A principal função do sistema vestibular está relacionada à manutenção da estabilidade do olhar e do equilíbrio por meio de mecanismos reflexos^(1)^. Este sistema detecta a aceleração angular e linear da cabeça em três dimensões e é responsável por gerar os reflexos vestíbulo-ocular (RVO) e o vestíbulo-espinal (RVE) que estabilizam a imagem visual na retina e ajustam a postura, respectivamente, durante a movimentação da cabeça. Além desse sistema, participam dessa função os sistemas visual, somatossensorial e proprioceptivo^(2-4)^.

A disfunção vestibular é uma condição de saúde que compromete as atividades de vida diária e acarreta acentuado prejuízo na qualidade de vida. Dentre os sintomas mais comuns da disfunção vestibular estão a vertigem ou outros tipos de tontura, alteração de marcha, quedas, perda auditiva e zumbido^(5,6)^.

Nas últimas décadas, o sistema vestibular tem sido visto não apenas como uma função reflexiva, e tem sido associado ao desempenho cognitivo^(1,7-11)^. Em geral, descobriu-se que lesões vestibulares bilaterais resultam em déficits cognitivos mais graves do que as disfunções unilaterais^(4,5,9)^. A perspectiva de cognição está relacionada a células corticais que unem conhecimentos sobre ações e percepções não apenas como veículos de ações integradas e processamento de percepções, mas também como uma base cerebral que possui ampla gama de funções corticais superiores, incluindo atenção, significados e conceitos, objetivos, sequências e intenções^(12)^_._

Achados laboratoriais e clínicos indicam a associação de entradas vestibulares com uma variedade de funções superiores, especialmente da memória, visto que esta também é determinada por outros processos cognitivos como a atenção, distúrbios emocionais e funções executivas^(13)^.

Bigelow e Agrawal^(1)^ listaram pesquisas que relacionam a disfunção vestibular a deficiência na capacidade visuoespacial, atenção, memória e função executiva. Estudos envolvendo aspectos cognitivos de pessoas com alterações ou queixas vestibulares comumente esbarram na escolaridade dos participantes^(14-18)^ como um fator confundidor dos resultados ou que não permitem uma generalização dos resultados. Considerando isso, este estudo incluiu apenas pessoas com no mínimo onze anos de estudo para verificar a relação entre o sistema vestibular e o desempenho cognitivo em adultos e idosos.

Dessa forma, esse estudo busca esclarecer a relação entre alterações vestibulares e o desempenho cognitivo de adultos e idosos escolarizados. Tendo em vista que indivíduos com alterações vestibulares podem ter algum grau de comprometimento cognitivo, este estudo busca analisar se alterações em nível periférico podem causar alterações em nível central (alterações cognitivas e de memória) e verificar se existe relação entre essas funções, em situações de desenvolvimento cognitivo favorecido pela escolaridade.

## MÉTODO

Trata-se de um estudo observacional do tipo transversal, de abordagem quantitativa, analítico e de observação individual. Esta pesquisa seguiu estritamente às recomendações éticas de pesquisa com seres humanos, tanto nacionais quanto internacionais, sendo aprovada por um Comitê de Ética em Pesquisa (parecer de aprovação número 4.857.581).

A amostra foi de conveniência, composta por 60 participantes com idade a partir de 19 anos, de qualquer gênero, com 11 ou mais anos de estudo formal e com audição dentro dos padrões de normalidade. Para garantir que todos os participantes fossem normo ouvintes foi realizada audiometria como critério de inclusão, devido ao fato de o comprometimento auditivo influenciar na compreensão das orientações da avaliação cognitiva e, consequentemente, no pior desempenho nas tarefas. A classificação dos limiares auditivos foi realizada de acordo com Lloyd e Kaplan^(19)^. Todos os participantes foram recrutados no Setor de Audiologia de uma clínica particular na cidade de Aracaju/SE e possuíam encaminhamento para realização dos exames.

Foram excluídos da pesquisa participantes com síndromes genéticas, em tratamento por quimioterapia, com diagnóstico de demência, com diagnósticos prévios de comprometimento cognitivo, com diagnóstico de hiperatividade ou déficit de atenção, com histórico de Acidente Vascular Encefálico, em uso de fármacos ansiolíticos, antidepressivos, anticonvulsivantes e sedativos; pessoas com deficiência auditiva de qualquer tipo ou grau e que fizeram reabilitação vestibular em algum momento.

Os participantes foram submetidos aos seguintes procedimentos: anamnese fonoaudiológica, exame *video Head Impulse Test* (vHIT), manobras de posicionamento de *Dix-Hallpike* e *Head Roll Test*, e provas cognitivas com Instrumento de Avaliação Neuropsicológica Breve NEUPSILIN.

A anamnese fonoaudiológica, aplicada pelo pesquisador, continha informações a respeito da saúde geral do indivíduo, dados audiológicos e otoneurológicos, além de informações sobre atividade ocupacional.

A avaliação otoneurológica foi dividida em duas etapas: realização do exame *vHIT e,* das manobras diagnósticas de posicionamento *Dix-Hallpike* e *Head Roll Test*. A manobra de *Dix-Hallpike* ^(20)^ avalia o deslocamento dos otólitos por meio da mudança de posição de cabeça, com maior sensibilidade para os canais semicirculares anteriores e posteriores. Foi realizada com o paciente sentado em uma maca, girando a cabeça 45º em direção à orelha a ser examinada e o deitando de forma que a cabeça estivesse inclinada aproximadamente 20º para fora do apoio da cabeça na posição supino. Durante a realização da manobra, o participante foi orientado a manter os olhos abertos para que fosse observada a possível ocorrência do nistagmo. Quando observado o nistagmo, considerou-se a manobra positiva. Quando não observado, a manobra foi considerada negativa.

O *Head Roll Test* ^(21)^ teve como objetivo avaliar o deslocamento dos otólitos através da mudança de posição de cabeça, sendo mais sensível para os canais semicirculares laterais. Foi realizado com o participante em decúbito dorsal em uma maca, com a cabeça elevada em 30º, de maneira que fosse possível realizar um giro de 90º para a orelha a ser examinada, sendo possível observar a possível ocorrência do nistagmo. Durante a realização da manobra, o participante foi orientado a manter os olhos abertos. A manobra foi considerada positiva quando observada a presença de nistagmo e, negativa, na ausência de nistagmo.

O vHIT^(22)^ avalia o reflexo vestíbulo-ocular em cada canal semicircular individualmente e em frequência fisiológica da aceleração angular da cabeça, por meio de impulsos cefálicos rápidos e de curta amplitude. Para a realização deste exame, foi utilizado o equipamento *EyeSeeCam vHIT* da marca *Interacoustics*. A avaliação foi realizada com o participante sentado em uma cadeira a um metro do alvo, posicionado à altura dos olhos, com uma máscara (óculos com uma câmera acoplada). Inicialmente foi realizada a calibração dos olhos e da cabeça e, posteriormente, a avaliação dos canais semicirculares laterais, seguido dos canais semicirculares verticais, de modo a avaliar os pares sinérgicos anterior direito/posterior esquerdo e anterior esquerdo/posterior direito. Durante a avaliação, o participante foi orientado a manter os olhos fixos no alvo, enquanto o examinador realizava os impulsos cefálicos referentes aos planos de estimulação dos seis canais semicirculares. Foram obtidos, ao menos, 15 impulsos para cada plano. Os movimentos cefálicos foram realizados com direção e frequência imprevisíveis, com baixa amplitude (10 à 20º) e velocidade entre 100 e 250º/s. Para cada impulso, foi fornecido o registro do movimento da cabeça e a resposta reflexa do olho mediante um gráfico sinusoidal. Esse registro permitiu o cálculo do ganho do RVO, que tem como valores de normalidade de 0,77 a 1,33 ms^(23)^. Os resultados do vHIT foram classificados como normal ou alterado e os relacionados à manobra de Dix-Hallpike em presente ou ausente.

A avaliação cognitiva foi realizada por meio do Neupsilin - Instrumento de Avaliação Neuropsicológica Breve^(24)^. Sua avaliação consiste em uma bateria de exame abreviado que tem como objetivo fornecer um perfil neuropsicológico (quantitativo e qualitativo). O instrumento avalia oito principais funções neuropsicológicas sendo composto 32 tarefas, com tempo médio de 50 minutos para aplicação total. Avalia as seguintes habilidades: orientação têmporo-espacial, atenção, percepção visual, memória (de trabalho, verbal episódica, semântica, visual de curto prazo e prospectiva), habilidades aritméticas, linguagem oral e escrita, praxias (ideomotora, construtiva e reflexiva) e funções executivas (resolução de problemas e fluência verbal fonêmico-ortográfica).

Esta avaliação foi realizada pelo examinador e, durante toda a aplicação, o participante estava confortavelmente sentado, com uma folha em branco e caneta em mãos para fazer anotações necessárias e responder às perguntas conforme orientado.

Para o cálculo do ponto de corte utilizado para a indicação de presença de déficit em alguma tarefa ou função foi utilizado o escore Z que permite a comparação dos resultados apesar da diferença de idade e escolaridade entre os participantes. De acordo com as organizadoras do Instrumento, este escore deve ser calculado levando em consideração o escore bruto do indivíduo, a média do grupo no teste (tarefa ou função) e o desvio-padrão do grupo no teste (tarefa ou função).

Dessa forma, o escore Z pode ser classificado em superior, quando está acima de 1,0; médio, quando está entre 0,99 e -0,99; alerta para déficit, com valores entre -1 e -1,4; déficit, com valor em -1,5; déficit moderado-severo, com valores entre -1,6 e -1,99 e déficit severo quando o valor está abaixo de -2.

Os dados foram analisados de forma descritiva e inferencial utilizando o software SPSS 25.0. Na análise descritiva das variáveis quantitativas, foram calculadas as medidas de tendência central (média e mediana), variabilidade (desvio-padrão) e posição (mínimo, máximo, primeiro e terceiro quartis). Na análise descritiva das variáveis qualitativas, foram calculadas a frequência absoluta e a frequência relativa percentual.

O escore Z foi calculado através da Fórmula 1:


Z=(X−M)/DP (1)

em que X é o valor obtido pelo indivíduo na tarefa, M é a média do grupo de referência (fornecida por idade e escolaridade pelo manual do teste), e DP é o desvio padrão desse grupo.

O teste de normalidade de Shapiro Wilk foi conduzido para a escolha entre o Teste de *T-Student* ou *Mann-Whitney*. Utilizou-se modelo de regressão linear múltipla para previsão das variáveis dependentes quantitativas. O método de seleção das variáveis independentes foi o *stepwise*. Foi considerado um nível de significância de 5% para todas as análises inferenciais.

## RESULTADOS

O estudo contou com 60 participantes com idades entre 19 e 74 anos, média de 49 anos e três meses, 40 mulheres (66,7%) e 20 homens (33,3%). Quanto à escolaridade, 30 participantes tinham o ensino médio completo e 30 participantes tinham o ensino superior completo.

O *vHIT* estava alterado em onze participantes sendo que as alterações observadas foram hipofunções de canal semicircular lateral e de canal semicircular posterior. Ainda, no teste do vHIT foram analisados os resultados alterados e não alterados em função do desempenho nas provas cognitivas (tabela 1). Observou-se diferença estatisticamente significativa para a atenção, percepção e habilidades aritméticas, com piores resultados em pessoas com vHIT alterado.

**Tabela 1:** Comparação das variáveis relacionadas ao desempenho cognitivo em participantes com e sem alteração do vHIT

Habilidade cognitiva		Estatística	Graus de liberdade	p
ORIENTAÇÃO TEMPOROESPACIAL	Teste U de Mann-Whitney	90,0		0,85
ATENÇÃO	Teste U de Mann-Whitney	35,5		0,01^*^
PERCEPÇÃO	Teste t de Student	-2,08	28,0	0,04*
MEMÓRIA DE TRABALHO	Teste t de Student	0,36	28,0	0,72
MEMÓRIA VERBAL	Teste t de Student	-0,96	28,0	0,34
MEMÓRIA DE LONGO PRAZO	Teste U de Mann-Whitney	80,0		0,50
MEMÓRIA DE CURTO PRAZO	Teste U de Mann-Whitney	82,5		0,58
MEMÓRIA PROSPECTIVA	Teste U de Mann-Whitney	90,5		0,87
HAB ARITMÉTICAS	Teste U de Mann-Whitney	47,5		0,03*
LINGUAGEM ORAL	Teste t de Student	1,32	28,0	0,20
LINGUAGEM ESCRITA	Teste U de Mann-Whitney	77,5		0,45
PRAXIAS	Teste t de Student	0,04	28,0	0,97
RESOLUÇÃO DE PROBLEMAS	Teste U de Mann-Whitney	92,0		0,93
FLUÊNCIA VERBAL	Teste t de Student	-0,12	28,0	0,91

* Indica valores estatisticamente significativos. vHit só esteve alterado em participantes do Grupo estudo (com queixas e/ou alterações vestibulares)

Nas manobras diagnósticas de posicionamento de *Dix-Hallpike*, em nove participantes a prova foi considerada positiva, ou seja, com nistagmo presente, e houve uma significante associação com piores desempenhos na função de orientação temporoespacial (Tabela 2). Enquanto que na prova *Head Roll Test* nenhum dos participantes apresentou nistagmo, ou seja, apresentaram a prova negativa.

**Tabela 2:** Comparação das variáveis relacionadas ao desempenho cognitivo em participantes, de acordo com a resposta da manobra de Dix-Hallpike

	Dix-Hallpike	Nº participantes	Média	Mediana	DP	p-valor
ORIENTAÇÃO TEMPOROESPACIAL	Ausente	51	0,2121	0,2963	0,821	0,564
	Presente	9	0,0122	0,558	1,542	0,034^*^
ATENÇÃO	Ausente	51	7,9744	7,0000	2,599	0,321
	Presente	9	9,2410	7,000	6,822	0,756
PERCEPÇÃO	Ausente	51	-0,1750	-0,0849	1,013	0,551
	Presente	9	-0,3956	-0,619	1,040	0,393
MEMÓRIA DE TRABALHO	Ausente	51	-0,1511	-0,0312	1,233	0,477
	Presente	9	-0,4823	-1,058	1,543	0,597
MEMÓRIA VERBAL	Ausente	51	-0,8146	-0,9789	1,056	0,112
	Presente	9	-1,4265	-1,310	1,005	0,159
MEMÓRIA DE LONGO PRAZO	Ausente	51	-0,5138	0,2174	1,550	0,853
	Presente	9	-0,6197	0,217	1,705	0,058
MEMÓRIA DE CURTO PRAZO	Ausente	51	-0,0491	0,3548	1,068	0,828
	Presente	9	-0,1349	0,481	1,204	0,232
MEMÓRIA PROSPECTIVA	Ausente	51	-0,7674	0,5306	1,683	0,267
	Presente	9	-1,4529	-2,130	1,749	0,743
HABILIDADES ARITMÉTICAS	Ausente	51	-1,3548	0,3158	2,488	0,314
	Presente	9	-0,4922	0,316	1,110	0,941
LINGUAGEM ORAL	Ausente	51	-0,7054	-0,9839	1,319	0,644
	Presente	9	-0,4885	-0,337	1,110	0,511
LINGUAGEM ESCRITA	Ausente	51	-0,9830	-0,7925	1,087	0,527
	Presente	9	-0,7381	-0,353	0,906	0,755
PRAXIAS	Ausente	51	-1,3552	-1,4928	1,454	0,882
	Presente	9	-1,4302	-1,769	0,866	0,934
RESOLUÇÃO DE PROBLEMAS	Ausente	51	-0,3997	0,3421	1,388	0,377
	Presente	9	-0,8523	0,429	1,522	0,574
FLUÊNCIA VERBAL	Ausente	51	-2,2948	-2,2495	0,350	0,626
	Presente	9	-2,2306	-2,249	0,437	0,975

* Indica diferença entre os grupos com nistagmo presente ou ausente no Dix-Hallpike

Legenda: DP = desvio padrão. Dix-Hallpike ausente ou presente, representa a presença ou não de nistagmo ao se realizar a manobra

Foi realizada a regressão linear múltipla para verificar se as variáveis independentes conseguem prever as variáveis dependentes, atenção, memória de trabalho, memória verbal, memória de longo prazo, memória de curto prazo, linguagem oral e linguagem escrita. Não houve modelos significativos para quatro variáveis dependentes: atenção, memória de curto prazo, linguagem oral e linguagem escrita.

A variável independente idade (β = -0,283; t = -2,249; p = 0,028) foi preditora da variável dependente memória de trabalho [F(1, 58) = 5,059; p=,028; R2 = 0,064]. O coeficiente negativo indica que, à medida que a idade aumenta, o desempenho em memória de trabalho diminui, ou seja, idades mais avançadas estão associadas a pior desempenho nessa função cognitiva. A equação que descreve essa relação é


(MEMÓRIA DE TRABALHO)=0,846+−0,021(Idade) (2)

conforme mostra a Tabela 3.

**Tabela 3:** Associação entre idade e desempenho em memória de trabalho

	Coeficientes não padronizados		Coeficientes padronizados	t	p-valor	95,0% IC para B	
	B	Erro padrão	Beta			Limite inferior	Limite superior
(Constante)	0,846	0,492		1,720	0,091	-0,138	1,831
IDADE	-0,021	0,009	-0,283	-2,249	0,028	-0,040	-0,002

Regressão linear múltipla; método stepwise

**Legenda:** IC = intervalo de confiança

Conforme a Tabela 4, as variáveis independentes DIX OD (β = -0,301; t = -2,463; p = 0,016) e VHIT (β = -0,25088778114266; t = -2,050; p = 0,044) foram previsoras da variável dependente memória verbal [F(1, 57) = 5,059; p=,028b; R2 = 0,0643]. Da mesma forma, o vHIT também teve um coeficiente negativo (B = -0,684; p = 0,045), sugerindo que alterações no reflexo vestíbulo-ocular estão associadas à redução no desempenho dessa função cognitiva. A equação que descreve essa relação é

**Tabela 4:** Associação entre alterações vestibulares (Dix-Hallpike OD e vHIT) e desempenho em memória verbal

	Coeficientes não padronizados		Coeficientes padronizados	t	p-valor	95,0% IC para B	
	B	Erro padrão	Beta			Limite inferior	Limite superior
(Constante)	-0,666	0,151		-4,410	0,000	-0,968	-0,363
DIX OD	-0,990	0,402	-0,301	-2,463	0,017	-1,795	-0,185
VHIT	-0,684	0,333	-0,251	-2,051	0,045	-1,351	-0,016

Regressão linear múltipla; método stepwise

**Legenda:** IC = intervalo de confiança; vHIT = Head Impulse Test; DIX = manobra de Dix-Hallpike; OD = orelha direita


$$\begin{array}{c} \text{(MEMÓRIA VERBAL})=-0,665+\\ -0,989(\text{DIX OD)}+-0,683(\text{VHIT)} \end{array}$$ (3)

A Tabela 5 mostra que a variável independente Idade (β = 0,358; t = 2,928; p = 0,004) foi preditora da variável dependente Memória de longo prazo [F(1, 58) = 8,574; R2 = 0,113]. A equação que descreve essa relação é

**Tabela 5:** Associação entre idade e desempenho em memória de longo prazo

	Coeficientes não padronizados		Coeficientes padronizados	t	p-valor	95,0% IC para B	
	B	Erro padrão	Beta			Limite inferior	Limite superior
(Constante)	-2,152	0,586		-3,675	0,001	-3,324	-0,980
IDADE	0,033	0,011	0,359	2,928	0,005	0,010	0,055

Regressão linear múltipla; método *stepwise*

**Legenda:** IC = intervalo de confiança


(MEMÓRIA DE LONGO PRAZO)=−2,151+0,032(Idade) (4)

## DISCUSSÃO

Os resultados deste estudo demonstram uma associação significativa entre alterações vestibulares, especificamente hipofunções do canal semicircular lateral e posterior observadas no vHIT; a presença de nistagmo no teste de Dix-Hallpike e o comprometimento de funções cognitivas em adultos e idosos. Essas descobertas reforçam a literatura que destaca o papel do sistema vestibular não apenas na regulação do equilíbrio e da postura, mas também como modulador do desempenho cognitivo, com possível impacto em áreas como a memória e a atenção.

A literatura da área aponta uma prevalência maior de tontura com o aumento da idade cronológica, com pico máximo entre 46 e 55 anos^(25-27)^. Em nossa população, a média de idade dos participantes foi de 49 anos e três meses, corroborando com os estudos citados.

Por se tratar de um estudo que avalia as questões cognitivas, a escolaridade dos indivíduos pode ser um fator que favoreça resultados enviesados, já que algumas teorias sugerem que existem diferenças nas conexões cerebrais de pessoas alfabetizadas e não alfabetizadas, de maneira que, o alto nível educacional está relacionado à reserva de capacidade neuronal, à maior quantidade de sinapses e vascularização cerebral. Sustentam ainda que a escolaridade é um dos fatores protetores contra o declínio cognitivo^(28-30)^.

Estudos nacionais e internacionais mostraram que a variável escolaridade tem efeito nas tarefas neuropsicológicas verbais e não verbais, assim como no desempenho de tarefas de memórias, atenção, linguagem e funções executivas^(14-18,31)^. Para este estudo, a escolaridade não se apresenta como uma limitação do estudo, já que tivemos o cuidado de que os indivíduos fossem pareados quanto ao nível de escolaridade. Além disso, todos os participantes apresentaram nível de escolaridade acima de 11 anos. Estudos com esta escolaridade podem contribuir para entender a cognição sem o viés da baixa escolaridade, possibilitando a compreensão da influência das alterações vestibulares na cognição, de uma forma mais direta, sendo este o grande diferencial do presente estudo.

O desempenho cognitivo foi influenciado pelas alterações evidenciadas pelo *vHIT*, *Dix-Hallpike* e pela idade. O *vHIT* alterado e *Dix-Hallpike* positivo indicam disfunção vestibular e influenciaram no desempenho das funções de orientação temporoespacial, atenção, percepção, habilidades aritméticas e memória verbal.

O *vHIT* alterado foi estatisticamente significativo nas funções atenção, percepção e habilidades aritméticas. De maneira que, quando o *vHIT* esteve alterado, o desempenho dessas funções foi pior. Os estudos de Bessot et al.^(32)^ e Popp et al.^(33)^ também demonstraram pior desempenho na função atencional em participantes com disfunção vestibular.

Não foi encontrada relação concisa entre a disfunção vestibular e o pior desempenho nas habilidades aritméticas na literatura, o que pode fomentar novos estudos^(34-36)^. Entretanto, a literatura mostra uma relação entre o pior desempenho nas habilidades aritméticas na população com alteração de atenção, o que pode estar relacionado ao achado deste estudo^(37-39)^.

A função de orientação temporoespacial também esteve relacionada ao pior desempenho nos participantes com disfunção vestibular pela presença de nistagmo na manobra de *Dix-Hallpike*. Estudos de Hufner et al.^(40)^, Guidetti et al.^(41)^, Xie et al.^(42)^, Ahmad et al.^(43)^ e Chari et al.^(11)^ corroboraram com este achado. Brandt et al. (2005)^(44)^ mostraram que há uma relação direta entre o tamanho do hipocampo e a orientação espacial. O estudo foi realizado através da volumetria de ressonância magnética, que demonstrou que participantes com disfunção vestibular bilateral desenvolveram uma atrofia significativa do hipocampo (em média diminuição de 16,9%) e apresentaram déficit de orientação espacial quando comparados ao grupo controle.

Neste estudo, observou-se que a memória verbal é pior naqueles indivíduos que possuem alteração no *vHIT* e/ou na manobra de *Dix-Hallpike*. Caixeta et al.^(45)^ encontraram essa mesma relação através da avaliação de 76 pacientes com disfunção vestibular periférica crônica. Entretanto, os instrumentos de avaliação do sistema vestibular não foram os mesmos do presente estudo e, para a avaliação cognitiva, utilizaram testes de rastreio (Mini Exame do Estado Mental, o Teste do Relógio e o Teste de Fluência Verbal). Assim, o presente estudo apresenta uma avaliação mais completa do desempenho cognitivo, haja visto que foi utilizada toda a bateria de teste que compõem o Neupsilin e não apenas testes de rastreio ou *screening*.

A variável idade mostrou influência na memória de longo prazo e na memória de trabalho, de forma que quanto maior a idade, pior o desempenho desta função, o que corrobora com diversos estudos realizados na literatura nacional e internacional^(31,46,47)^. Um estudo realizado com 1126 participantes com idade entre 60 e 100 anos avaliou o efeito da idade na memória. Os autores utilizaram o teste *Mini-Mental State Examination* que avalia a memória de trabalho; o teste *Montreal Cognitive Assessment* que avalia a memória declarativa verbal; e o teste Figura Complexa de Rey-Osterrieth que avalia a memória visuoespacial. Assim, eles observaram que a idade influencia em todos os tipos de memórias avaliadas, corroborando, em parte, com o nosso achado que aponta a influência da idade na memória de trabalho^(48)^. Talvez a escolaridade dos nossos participantes tenha contribuído para menores perturbações em outros tipos de memória, pois todos tinham ao menos 11 anos de estudo formal.

Uma das limitações deste estudo refere-se ao tamanho da amostra, composta por conveniência, restringindo a generalização dos resultados para a população em geral. Recomenda-se, portanto, a realização de estudos futuros com amostras maiores e representativas, a fim de permitir inferências mais abrangentes sobre a população brasileira.

## CONCLUSÃO

A presença de alterações vestibulares esteve relacionada à piores desempenhos cognitivos de adultos e idosos nas provas de orientação temporoespacial, atenção, percepção, habilidades aritméticas e memória verbal. O aumento da idade foi associado ao pior desempenho na memória de trabalho e na memória de longo prazo.
